# Extracellular vesicles from maternal uterine cells exposed to risk factors cause fetal inflammatory response

**DOI:** 10.1186/s12964-021-00782-3

**Published:** 2021-10-07

**Authors:** Megan C. Shepherd, Enkhtuya Radnaa, Ourlad Alzeus Tantengco, Talar Kechichian, Rheanna Urrabaz-Garza, Ananth Kumar Kammala, Samantha Sheller-Miller, Ramkumar Menon

**Affiliations:** 1 Division of Maternal-Fetal Medicine and Perinatal Research, Department of Obstetrics and Gynecology, The University of Texas Medical Branch at Galveston, 301 University Blvd., Galveston, TX 77555-1062 USA; 2 Department of Biochemistry and Molecular Biology, College of Medicine, University of the Philippines Manila, Manila, Philippines

**Keywords:** Cigarette smoke, Communication, Cytokines, Exosomes, Inflammation, Oxidative stress, Pregnancy, Preterm birth, Signaling

## Abstract

**Background:**

Fetal cell-derived exosomes (extracellular vesicles, 40–160 nm) are communication channels that can signal parturition by inducing inflammatory changes in maternal decidua and myometrium. Little is known about maternal cell-derived exosomes and their functional roles on the fetal side. This study isolated and characterized exosomes from decidual and myometrial cells grown under normal and inflammatory/oxidative stress conditions and determined their impact on fetal membrane cells.

**Methods:**

Decidual and myometrial cells were grown under standard culture conditions (control) or exposed for 48 h to cigarette smoke extract or tumor necrosis factor-α, as proxies for oxidative stress and inflammation, respectively. Exosomes were isolated from media (differential ultra-centrifugation followed by size exclusion chromatography), quantified (nano particle tracking analysis), and characterized in terms of their size and morphology (cryo-electron microscopy), markers (dot blot), and cargo contents (proteomics followed by bioinformatics analysis). Maternal exosomes (10^9^/mL) were used to treat amnion epithelial cells and chorion trophoblast cells for 24 h. The exosome uptake by fetal cells (confocal microscopy) and the cytokine response (enzyme-linked immunosorbent assays for IL-6, IL-10, and TNF-α) was determined.

**Results:**

Exosomes from both decidual and myometrial cells were round and expressed tetraspanins and endosomal sorting complexes required for transport (ESCRT) protein markers. The size and quantity was not different between control and treated cell exosomes. Proteomic analysis identified several common proteins in exosomes, as well as unique proteins based on cell type and treatment. Compared to control exosomes, pro-inflammatory cytokine release was higher in both amnion epithelial cell and chorion trophoblast cell media when the cells had been exposed to exosomes from decidual or myometrial cells treated with either cigarette smoke extract or tumor necrosis factor-α. In chorion trophoblast cells, anti-inflammatory IL-10 was increased by exosomes from both decidual and myometrial cells.

**Conclusion:**

Various pathophysiological conditions cause maternal exosomes to carry inflammatory mediators that can result in cell type dependent fetal inflammatory response.

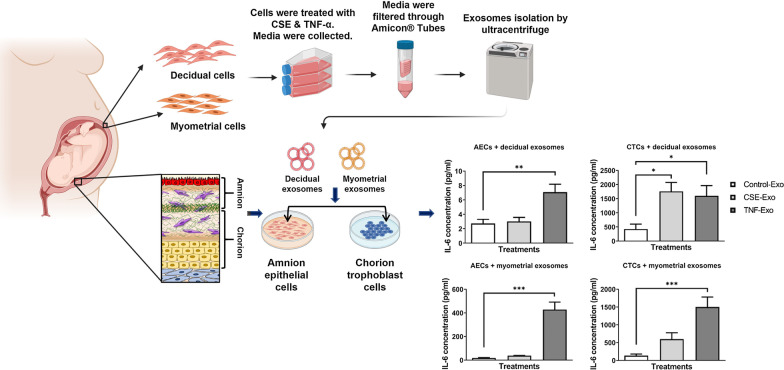

**Video Abstract**

**Supplementary Information:**

The online version contains supplementary material available at 10.1186/s12964-021-00782-3.

## Background

Physiologic adaptations required to maintain a healthy pregnancy requires persistent communication between the fetus and the mother [1]. Maternal–fetal communication is also essential for properly timing the initiation of parturition [2, 3]. Multiple mediators are used for this purpose, including endocrine factors [4], immune cells [5], cytokines [6], and mechanical factors [7]. Recently, extracellular vesicles (EV) have been implicated in performing communication functions [8]. EVs are circular vesicles that are surrounded by a lipid bilayer, lack nuclei and cannot replicate [9]. EVs are released by both maternal and fetal cells and carry various cargo that are reflections of the physiologic and metabolic state of the cells at the time of their release [10]. EV cargo contents can be taken up by recipient cells to induce specific functional effects [11]. Functional contributions of EVs are implicated in various aspects of reproduction, including embryogenesis [12, 13], implantation [14–16], pregnancy, and parturition [17–20].

Recent reports have referred to EVs primarily as ectosomes and exosomes, based on their size and how they are formed in a cell [21, 22]. Ectosomes pinch off the surface of the plasma membrane via outward budding and include microvesicles in the size range of ~ 50 nm to 1 mm. Exosomes are particles with a size range of approximately 40–160 nm and are of endosomal origin [22]. The functions of exosomes are well reported in reproductive and perinatal biology, and include acting as communication signals, biomarkers of various pregnancy pathologies and as drug delivery vehicles during pregnancy [8, 18]. Using both in vitro and in vivo models, we have reported the following regarding exosome function: (1) fetal exosomes can traverse through uterine tissue barriers in animal models of pregnancy [23]; (2) senescent fetal membrane cell-derived exosomes cause maternal uterine cell inflammation [11]; (3) injection of inflammatory cargo-enriched exosomes cause preterm birth in animal models [24]; and 4) fetal (placental) exosomes are predictive markers of high risk pregnancies [17, 25].

To further prove that exosome trafficking is bidirectional during pregnancy, we developed a transgenic animal model containing a membrane-targeted red fluorescent protein (tdTomato), which switches to green fluorescent protein when exposed to cyclic recombinase (Cre) [26]. Using this model, we showed ~ 35% of exosomes isolated from maternal plasma at term were fetal in origin, and that fetal exosomes were also localized in maternal uterine tissues. Likewise, injected maternal Cre-enriched exosomes crossed the placenta, and caused green fluorescent protein expression in fetal cells. Furthermore, green fluorescent protein-positive exosomes released from fetal cells were isolated from maternal blood [26]. These studies supported bidirectional trafficking and a functional impact of both fetal and maternal exosomes on opposite sides during pregnancy.

Although exact mechanisms are not fully elucidated, constitutive exosome-based signaling between maternal and fetal tissues are expected to provide physiologic signals needed for pregnancy maintenance in recipient cells. Alterations to these signals, primarily influenced by changes in their cargo due to various risk exposures, can compromise tissue homeostasis required to maintain pregnancy. Multiple reports have shown that risk exposures cause exosome cargo changes in human fetal membrane and placental tissue-derived exosomes that are capable of eliciting distinct effects in maternal cells [27–29]. Currently no data exists to show that risk exposure on the maternal side (e.g. infection or inflammation) during pregnancy can produce exosomes with specific cargo that can lead to pathologic changes on the fetal side. Therefore, we hypothesize that maternal risk exposures during pregnancy can generate inflammatory cargo-enriched exosomes from uterine cells that can reach fetal tissues and cause a fetal inflammatory response associated with adverse pregnancy outcomes. To test the hypothesis, we treated decidual and myometrial cells with cigarette smoke extract (CSE) and tumor necrosis factor (TNF)-α, as proxies for oxidative stress (OS) and inflammation, respectively [30–32]. CSE treatment of cells has been shown to mimic intrauterine OS seen during term labor and in spontaneous preterm birth, either with or without rupture of the membranes. Exosomes were isolated from culture media samples, then characterized and the proteomic cargo contents analyzed. Fetal amnion epithelial cells (AEC) and chorion trophoblast cells (CTC) were exposed to exosomes from treated and untreated maternal cells, and inflammatory cytokine responses were determined. We report that OS and inflammation cause decidual and myometrial cells to produce exosomes with distinct cargo that is capable of producing a differential fetal inflammatory response. This can be associated with pathways leading to adverse pregnancy outcomes, like preterm birth.

## Methods

### IRB approval

This study protocol was approved by the Institutional Review Board at the University of Texas Medical Branch (UTMB) at Galveston, TX, as an exempt protocol to use discarded placentas after normal term cesarean deliveries (UTMB 11-251). No subject recruitment or consenting was done for this study and no identifiers were collected.

Immortalized decidual, myometrial and amnion epithelial cell lines were used. Chorion trophoblast cells were primary cells harvested from discarded placentas from the exempt protocol listed above. Placentas were collected from women age 18–40 years old undergoing elective repeat cesarean delivery, between 37 and 41 weeks gestation, prior to spontaneous labor. Women were excluded if they had a history of preterm labor or delivery, premature rupture of membranes, preeclampsia, placental abruption, fetal growth restriction, gestational diabetes, Group B streptococcus carrier status, history of treatment for a urinary tract infection during pregnancy, sexually transmitted infections during pregnancy, chronic infections such as HIV and hepatitis, or a history of cigarette smoking or drug and alcohol abuse.

Decidual and amnion epithelial cells were immortalized by standard HPV16 E6E7 retrovirus protocol as described previously [33]. Cells were passaged at least 10 times before the experiments.

### Myometrial cell culture

Myometrial cells were obtained from the hTERT-HM ^A/B^ myometrial cell line (a gift from Dr. Sam Mesiano, Case Western Reserve University, Cleveland, OH) [34]. The myometrial cells were plated in T75 flasks and cultured in media containing Dulbecco’s Modified Eagle Medium (DMEM, phenol red free, Corning®, #17-205-CV, Corning, NY, USA), supplemented with 10% heat inactivated fetal bovine serum (FBS, Sigma, F2442, St. Louis, MO, USA), 0.5% penicillin/streptomycin (Sigma-Aldrich), 2 mM L-glutamine, 100 µg/mL Geneticin™ (G418 Sulfate, Gibco, # 10131035, Franklin Lakes, NJ, USA), 1 µg/mL Hygromycin B (Invitrogen, #10687-010, Carlsbad, CA, USA), and 5 µg/mL Blaticidin S HCl (Invitrogen, #R210-01). In addition to the antibiotics listed, the treatment media was also supplemented with doxycycline (DOX, MilliporeSigma, #324385, 100 ng/mL) and Genostat Ligand (GSL, 100 nM) to increase PR-A and PR-B expression, respectively. Cells were grown at 37 °C, 95% air humidity and 5% CO_2_ until they reached 80% confluence.

### Decidual cell culture

Immortalized decidual cells were grown in media containing complete DMEM/F12 (Gibco, #10–092-CV) media plus 10% heat inactivated FBS (Sigma, F2442), 1% penicillin/streptomycin, 1% amphotericin B (Sigma, A2942) and 10 ng/mL epidermal growth factor (Sigma, E4127) at 37 °C, 95% air humidity and 5% CO_2_ until 80% confluence was reached.

### Amnion epithelial cell culture

Immortalized AECs were grown in T75 flasks with complete Keratinocyte Serum Free Media (KSFM – Gibco, #17005042) supplemented with human recombinant epidermal growth factor (rEGF, #10450-013, 2.5 µg), bovine pituitary extract (BPE, #13028-014, 25 mg) and primocin (Invitrogen, Cat #cat-pm-1, 50 mg/mL) and incubated at 37 °C, 95% air humidity and 5% CO_2_ until 80% confluence was reached.

### Isolation and culture of chorion trophoblast cells (CTC)

CTC cultures were performed as described in our prior report [35] and growing in the complete media (DMEM/F12, Gibco) supplemented with 10% FBS (Sigma-Aldrich), 100 U/mL penicillin G, 100 mg/mL of streptomycin (Sigma-Aldrich), 1.0 mg/mL of amphotericin B (Sigma-Aldrich), and trophoblast growth supplement (ScienCell, #7152, Carlsbad, CA, USA)at 37 °C, 95% air humidity and 5% CO2 to 80% confluence. The primary CTCs were isolated 7 different times for the experiments (N = 7).

### Decidual and myometrial cells under normal and inflammatory/OS cell culture conditions

Treatment media was the same as described above, with the exception of using exosome-free media. This was accomplished by ultra-centrifuging the 10% FBS for 16 h at 100,000*g*. Following ultra-centrifugation, the FBS supernatant was collected, and the pellet was discarded. The supernatant was sterilized by passing it through a 0.22 μm Steriflip filter unit.

CSE was used to induce OS in decidual and myometrial cells. A commercial cigarette was lit, and the smoke was infused into 25 mL of exosome-free media. The CSE stock was then sterilized by passing it through a 0.22 μm Steriflip filter unit. The stock CSE was diluted 1:50 in exosome free media prior to use.

TNF-α (MilliporeSigma, # T6674) was used as a proxy for inflammation. TNF-α was added to exosome-free media to give a final concentration of 50 ng/mL. When the decidual and myometrial cells reached 80% confluence, their flasks were rinsed with sterile 1 × PBS. Next, the cells were treated with exosome-free cell media (control conditions) or with exosome-free media either containing CSE (OS conditions) or TNF-α (inflammatory conditions), at 37 °C, 95% air humidity and 5% CO_2_, for 48 h. The culture media from control, OS, and inflammatory conditions were collected after treatment and stored at − 80 °C.

### Exosome isolation from culture media

Prior to exosome isolation, cell supernatant media was thawed overnight. Exosomes were isolated using differential ultra-centrifugation as described previously [27, 36–38]. The isolated exosomes were stored at − 80 °C until needed.

### Exosome characterization

We used multiple approaches to characterize exosomes from decidual and myometrial cells: cryo-electron microscopy (shape), nanoparticle tracking analysis using ZetaView (size and quantity), and dot blot (markers).

### Cryo-electron microscopy

Cryo-electron microscopy was performed as described previously [23, 24].

### Nanoparticle tracking analysis with ZetaView

Nanoparticle tracking analysis was performed using the ZetaView PMX 110 (Particle Metrix, Meerbusch, Germany) and its corresponding software (ZetaView 8.05.05) [39]. Frozen exosomes were thawed on ice. A 1:50 dilution of the exosome sample was made with PBS. Samples containing exosomes from control, CSE or TNF-α treatments were loaded in the ZetaView Nanoparticle Tracking Analyzer and the number of particles/mL and the size distribution was counted for each sample.

### Dot blot analysis

Dot blot analysis was performed as per the manufacturer’s instruction for the Exo-Check Antibody array (System Biosciences), which included 12 pre-printed spots: 8 known exosome markers (CD63, CD81, ALIX, FLOT1, ICAM1, ANXA5, and TSG101) and 4 controls (2 positive controls, 1 negative control and GM130). GM130, a cis-Golgi marker, monitors any cellular contamination in the exosome isolation.

### EV-track

We have submitted all relevant data of our experiments to the EV-TRACK knowledgebase (EV-TRACK ID: EV210163) [40].

### Proteomic analysis of maternal plasma exosomes by mass spectrometry

#### Exosome protein clean up and digestion

The protein profile of exosomes was established by liquid chromatography (LC) and mass spectrometry (MS). To make a starting volume, 25 μg of exosomes were diluted in 10% SDS in 0.1 M triethylammonium bicarbonate (TEAB), so that the dilute sample had a final concentration of 5% SDS. The samples were then sonicated on ice for 10 min. Then, 1 μL of 0.25 M tris(2-carboxyethyl)phosphine (TCEP) per 25 μL of starting volume was added to each tube, to achieve a final concentration of 0.01 M. The samples were incubated for 1 h at 55 °C. The samples were cooled before 1 μL of 20 mM iodoacetamide in double distilled water, per 25 μL starting volume, was added to each tube. The samples were incubated for 45 min in the dark. The samples were then transferred to tubes containing 8 M dry urea and vortexed. Phosphoric acid (12%) was added to give a final concentration of 1.2%. Next, 165 μL of S-Trap buffer (90% MeOH, 100 mM TEAB, pH 7.5) was added to the acidified lysate, for every 25 μL starting volume. The lysate was added to the S-Trap microcolumn (Protifi, Huntington, NY) and centrifuged at 4000*g* for 2 min. The flow-through was discarded and the column was washed with 150 μL of S-Trap buffer, before being centrifuged at 4000*g* for 2 min, 3 times. The columns were transferred to new tubes and incubated with 1.25 μg trypsin in 25 μL of 50 mM TEAB for 2 h at 47 °C. Proteins were eluted by centrifugation at 4000*g* for 2 min with 50 mM TEAB, 0.2% formic acid, 50% acetonitrile/0.2% formic acid, and finally 80% acetonitrile/0.1% formic acid. The final elute was dried.

#### Nanoscale liquid chromatography coupled to tandem mass spectrometry analysis

Peptide mixtures were analyzed by nanoflow liquid chromatography-tandem mass spectrometry (nano-LC–MS/MS) using a nano-LC chromatography system (UltiMate 3000 RSLCnano Dionex, Thermo Fisher Scientific, San Jose, CA). The nano-LC–MS/MS system was coupled to a Thermo Orbitrap Fusion mass spectrometer (Thermo Fisher Scientific, San Jose, CA) through a nanospray ion source (Thermo Scientific). A trap and elute method was used to desalt and concentrate the sample, while preserving the analytical column. The trap column (Thermo Scientific) was a C18 PepMap100 (100 μm × 20 mm, 5 µm particle size), while the analytical column was an Acclaim PepMap 100 (75 μm X 25 cm, Thermo Scientific). After equilibrating the column in 98% solvent A (0.1% formic acid in water) and 2% solvent B (0.1% formic acid in acetonitrile (ACN)), the samples (5 µL in solvent A) were injected onto the trap column. They were subsequently eluted (300 nL/min) by gradient elution onto the C18 column as follows: isocratic at 2% B, 0–5 min; 2% to 6% B, 5–6 min; 6% to 32% B, 6–65 min; 32% to 50% B, 49–50 min; 50% to 90% B, 71–72 min; isocratic 90% B, 72–73 min; 90% to 5% B, 73–74 min; isocratic 5% B, 74–74.5 min; 5% to 90% B, 74.5–75 min; isocratic 90% B, 75–76 min; 90% to 2% B, 76–77 min; and isocratic 2% B, 77–90 min.

All LC–MS/MS data was acquired using XCalibur, version 4.7.73.11 (Thermo Fisher Scientific), in positive ion mode, using a top speed data-dependent acquisition (DDA) method with a 3 s cycle time. The survey scans (m/z range of 375–1500) were acquired in the Orbitrap at 120,000 resolution (at m/z = 400) in profile mode, with a maximum injection time of 50 ms and an automatic gain control (AGC) target of 400,000 ions. The S-lens RF level was set to 60. Isolation was performed in the quadrupole with a 1.6 Da isolation window, and CID MS/MS acquisition was performed in profile mode using a rapid scan rate, with detection in the ion-trap using the following settings: parent threshold = 5,000; collision energy = 35%; maximum injection time = 35 ms; and an AGC target of 2,000 ions. Monoisotopic precursor selection (MIPS) and charge state filtering were on, with charge states 2–7 included. Dynamic exclusion was used to remove selected precursor ions, with a ± 10 ppm mass tolerance, for 60 s after acquisition of one MS/MS spectrum.

#### Database searching

Tandem mass spectra were extracted and charge states deconvoluted using Proteome Discoverer (Thermo Fisher, version 2.4.1.15). Deisotoping was not performed. All MS/MS spectra were searched against the Uniprot Human database (reviewed June 11, 2019), using Sequest. Searches were performed with a parent ion tolerance of 10 ppm and a fragment ion tolerance of 0.60 Da. Trypsin was specified as the enzyme, allowing for two missed cleavages. Fixed modifications of carbamidomethyl (C), and variable modifications of oxidation (M) and deamidation (N, Q), were specified in Sequest.

### Ingenuity pathway analysis (IPA) of identified proteins

Pathway enrichment analyses were performed with Ingenuity pathway analysis (, Qiagen, Hilden, Germany) using Fisher’s exact test. IPA was performed to identify canonical pathways, based on fold change and z scores. Significantly enriched pathways for the proteins and scenarios were identified using P < 0.01.

All differentially expressed proteins data can be accessed at: https://figshare.com/articles/dataset/Decidua_and_myometrial_cell_exosome_proteomics_data/14622828/1.

### Immunofluorescence staining of exosomes and fluorescence microscopy to localize exosomes in recipient cells

Isolated control, CSE and TNF-α exosomes from both decidual and myometrial cells were labeled with DiOC18 (3,3′-Dioctadecyloxacarbocyanine Perchlorate (DiO), D275, Invitrogen, Carlsbad, CA) by resuspending the final exosome pellet in 100 μL of 100 μM DiO. The exosomes were then incubated at 37 °C for 30 min. After the incubation step, 300 μL of 5% bovine serum albumin (BSA) was added to the DiO-labeled exosomes. To remove excess DiO, the exosome suspension was transferred to Amicon Ultra-15 Centrifugal Filter Units (UFC910024, 100 kDa Merk Millipore Ltd., Tullagreen, Carrigtwohill, Co. Cork, Ireland) and centrifuged at 4000*g* for 10 min. The retentate was used for the exosome uptake assay in recipient cells. AECs and CTCs were plated on glass cover slips at a density of 50,000 cells/slip and incubated overnight prior to the treatment with the exosomes. The labeled exosomes were then added to the cover slip and incubated at 37 °C for 4 h. The cells were then fixed with 4% paraformaldehyde, permeabilized with 0.5% Triton X, and blocked with 3% BSA in PBS. For counter staining to visualize cell morphology, cells were incubated overnight at 4 °C with primary antibodies against cytokeratin-7 (CK-7, 1 μL/mL, ab9021, Abcam) for chorion trophoblast cells or cytokeratin-18 (CK-18, 1 μL/mL, ab668, Abcam) for amnion epithelial cells. After washing with PBS, the slides were incubated with Alexa Fluor 594-conjugated secondary antibodies (Life Technologies, Carlsbad, CA) diluted 1:400 in PBS, for 1 h. The slides were then washed with PBS and treated with 4′,6-diamidino-2-phenylindole (DAPI). Following this, the slides were mounted using MOWIOL mounting medium. The slides were dried overnight then imaged using a Keyence BZ-X810 all-in-one fluorescence microscope (20x). Uniform laser settings, brightness, contrast, and collection settings were matched for all images collected. Z-stacks were generated from images taken at 0.25–0.4 µm intervals. ImageJ software, version 1.51J (NIH, Bethesda, MD; http://imagej.nih.gov/ij), was used to visualize z-stacks and confirm the location of the exosomes in relation to the cells. Three-dimensional reconstructions of the cells were created using IMARISViewer software, version 9.5.1 (Bitplane, Concord, MA, USA), to further confirm the location of the exosomes in relation to the target cells.

### Exosome treatments of cells

AECs and CTCs were placed in 6-well plates at a concentration of 500,000 cells/well and grown overnight. The following day, the cell media was removed, the cells were washed with PBS and the media was replaced with treatment media. Exosomes from control, CSE and TNF-α groups were placed in exosome-free media at a concentration of 2 × 10^9^ exosomes/well. The cells were incubated with the exosomes for 24 h. A negative control was used that included exosome-free media only, and 2 positive controls were used: CSE at 1:50 and TNF-α at 50 ng/mL. When the treatment was complete, the media was collected from each well and stored at − 80 °C. This was repeated a total of 7 times. The cells were then collected from the wells. To collect the cells, each well was treated with radio immunoprecipitation assay buffer, which contained phenylmethanesulfonyl fluoride (Fluka), protease inhibitor cocktail (Sigma-Aldrich) and Halt phosphatase inhibitor cocktail (Thermo-Scientific), and the cells were manually scraped from the well using a cell scraper. The cells were placed on ice for 10 min, vortexed for 10 s, sonicated for 30 s, vortexed for an additional 10 s and then placed on ice for 10 min. The lysed cells were then flash frozen using liquid nitrogen and stored at − 80 °C.

### Exosome blocking experiments

To determine if the effects in recipient cells were mediated by exosomes, a control experiment was performed that consisted of a cold incubation. For this, the exact treatments, as explained in the last section, were done with one change – after treating the cells with the exosomes, they were incubated at 4 °C for 6 h.

### Enzyme-linked immunosorbent assay for determining inflammatory marker responses

The media collected from the exosome treatments and exosome blocking treatments were analyzed using an enzyme-linked immunosorbent assay (ELISA) for common inflammatory mediators: interleukin (IL)-6, IL-10 and TNF-α. The ELISA was performed after the media was thawed and spun down to remove cells and other debris. The media was pipetted into the ELISA plate wells, as per kit instructions (R&D Systems-Quantikine ELISA). The results of the ELISA were obtained using a Synergy H4 microplate reader (BIO-TEK).

### Statistical analysis

The distribution of inflammatory markers was compared between exosome treated cells from normal conditions, CSE conditions, or TNF-α conditions using one-way analysis of variance (ANOVA). These analyses were conducted for each cell type. A *P* value of < 0.05 was considered to be statistically significant.

## Results

**Fig. 1:**
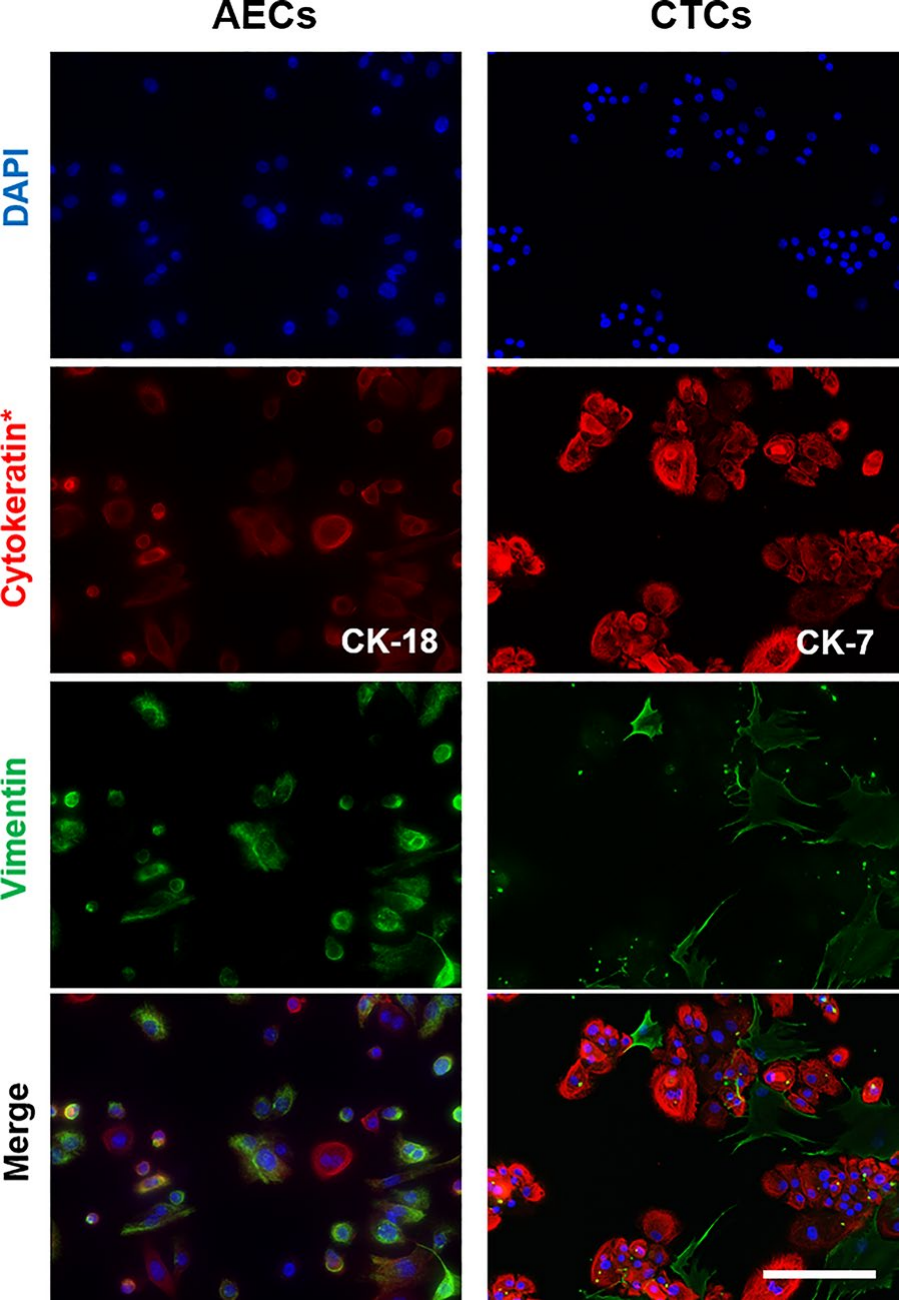
Representative images of immunocytochemical staining for the marker proteins of CTCs and AECs (N = 3). CK-18 = cytokeratin-18; CK-7 = cytokeratin-7. Scale bar = 100 µm

Prior to proceeding with our experiments, we confirmed morphology and presence of cell-specific markers on each of the fetal cell types using immunofluorescent staining of specific cell markers (Fig. 1).

### Exosome quantity and character are not altered by cell or stimulus type

**Fig. 2:**
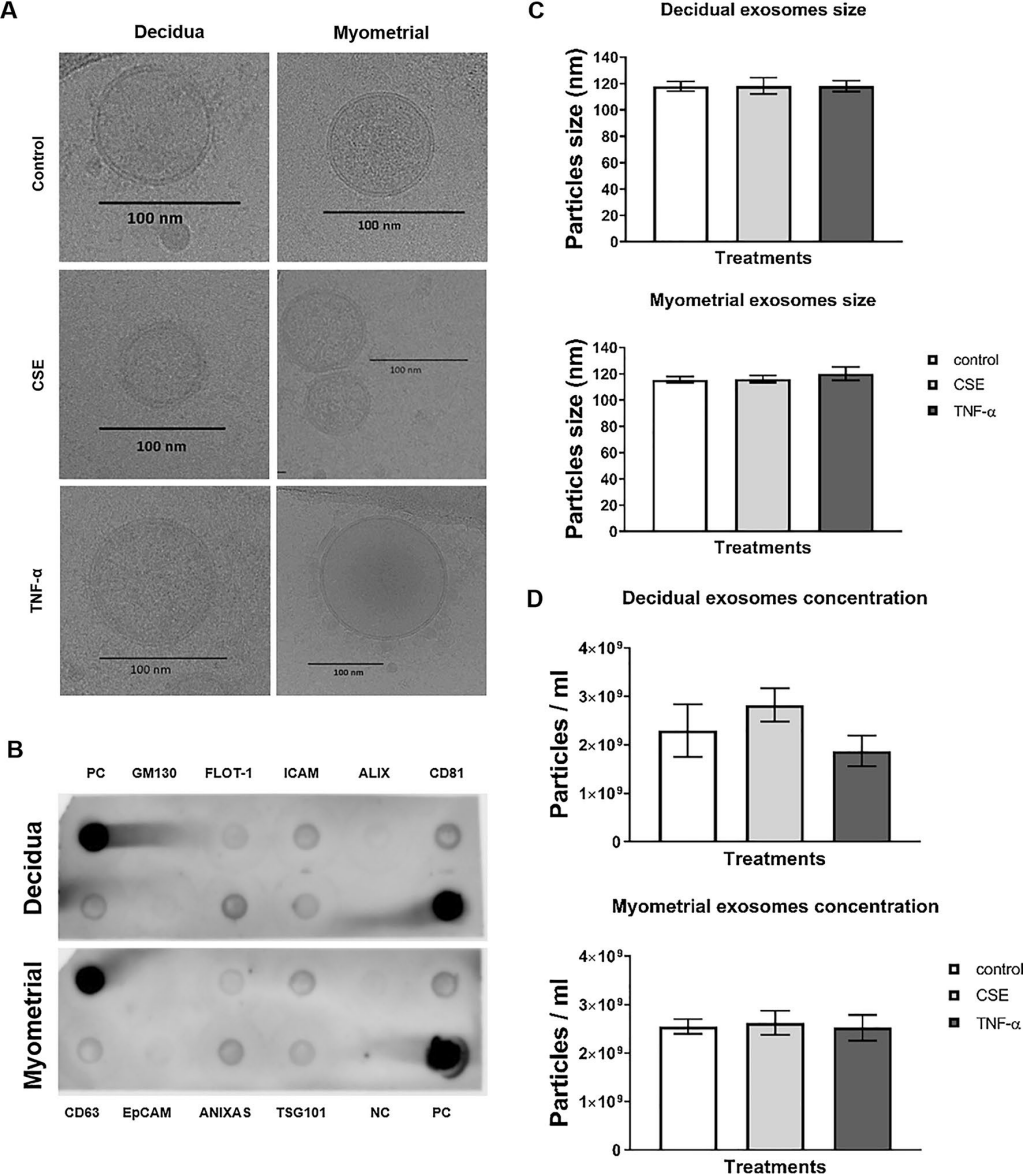
Characterization of decidual and myometrial cell-derived exosomes. **A** Representative cryo-electron microscope images of control, CSE- and TNF-α-treated decidual and myometrial cell-derived exosomes (N = 3). **B** Dot blot analysis showing the detection of exosome marker proteins in the decidual and myometrial cell-derived exosome using 50 µg of total protein lysates. PC = positive control; NC = negative control. **C** Nanoparticle tracking analysis (NTA) showing control, CSE- and TNF-α-treated decidual and myometrial cell-derived exosome sizes (N = 4). **D** Nanoparticle tracking analysis (NTA) showing control, CSE- and TNF-α-treated decidual and myometrial cell-derived exosome concentrations (N = 4). Data represented as mean ± SEM with one-way analysis of variance (ANOVA)

The size and quantity of exosomes was determined using cryo-electron microscopy and ZetaView analysis, respectively. Cryo-electron microscopy of exosomes isolated from control and conditioned media round exosomes, with a double membrane that ranged from 106.9 to 134.8 nm regardless of cell or treatment type (Fig. 2A). Decidual cells produced an average of 2.3 × 10^9^ particles/mL under normal cell culture conditions, 2.83 × 10^9^ particles/mL under OS conditions induced by CSE treatment, and 1.88 × 10^9^ particles/mL under inflammatory conditions after TNF-α treatment. The average sizes of decidual exosomes from control, CSE and TNF-α groups were 117.9 nm, 118.3 nm and 118.1 nm, respectively (Fig. 2C and D). Myometrial cells produced an average of 2.55 × 10^9^ particles/mL under normal cell culture conditions, 2.63 × 10^9^ particles/mL after CSE treatment, and 2.53 × 10^9^ particles/mL after TNF-α treatment. The average sizes of myometrial cell-derived exosomes from control, CSE and TNF-α groups were 115.6 nm, 116.1 nm and 120.1 nm, respectively (Fig. 2C and D). The average size of exosomes, regardless of cell type or conditions, confirmed the homogeneity of the exosome preparations used for the rest of our experiments. Using dot blot analysis, we determined that exosomes from both decidual and myometrial tissues contained tetraspanin markers CD63, CD81, FLOT-1, ICAM, ANXAS, TSG101, ALIX and EpCAM, but did not contain marker GM130, further ruling out any cellular organelle or other extracellular vesicle contamination (Fig. 2B).

### Exosome protein cargo displays differential expression

**Fig. 3:**
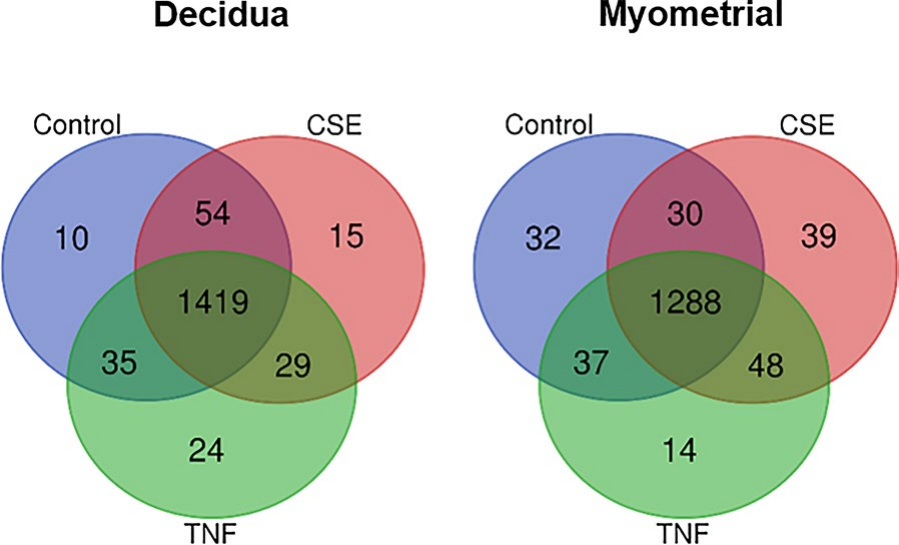
Venn diagram of exosomal protein cargos in control, CSE- and TNF-α-treated decidual and myometrial cell-derived exosomes detected by proteomic analysis. In decidual exosomes, 1419 proteins were common to all treatments, 10 proteins were unique to control samples, 15 proteins were unique to CSE-treated samples, and 24 proteins were unique to TNF-α-treated samples. In myometrial exosomes, 1288 proteins were common to all treatments, 32 proteins were unique to control samples, 39 proteins were unique to CSE-treated samples, and 14 proteins were unique to TNF-α-treated samples

Proteomics analysis showed over 1500 proteins in exosomes derived from control, CSE and TNF-α-treated decidual and myometrial cells. To further clarify the distributions of differentially expressed proteins in exosomes under specific conditions, Venn diagrams were created to show unique and shared cargo proteins. The differential expression of exosomal protein cargo is shown based on cell specific responses to control, CSE and TNF-α conditions. Decidual exosomes had a total of 1419 proteins common to all treatment groups. Control, CSE and TNF-α groups had 10, 15 and 24 unique proteins, respectively. Myometrial exosomes had a total of 1288 proteins that were common to all treatment groups. Control, CSE and TNF-α groups had 32, 39 and 14 unique proteins, respectively (Fig. 3).

**Fig. 4:**
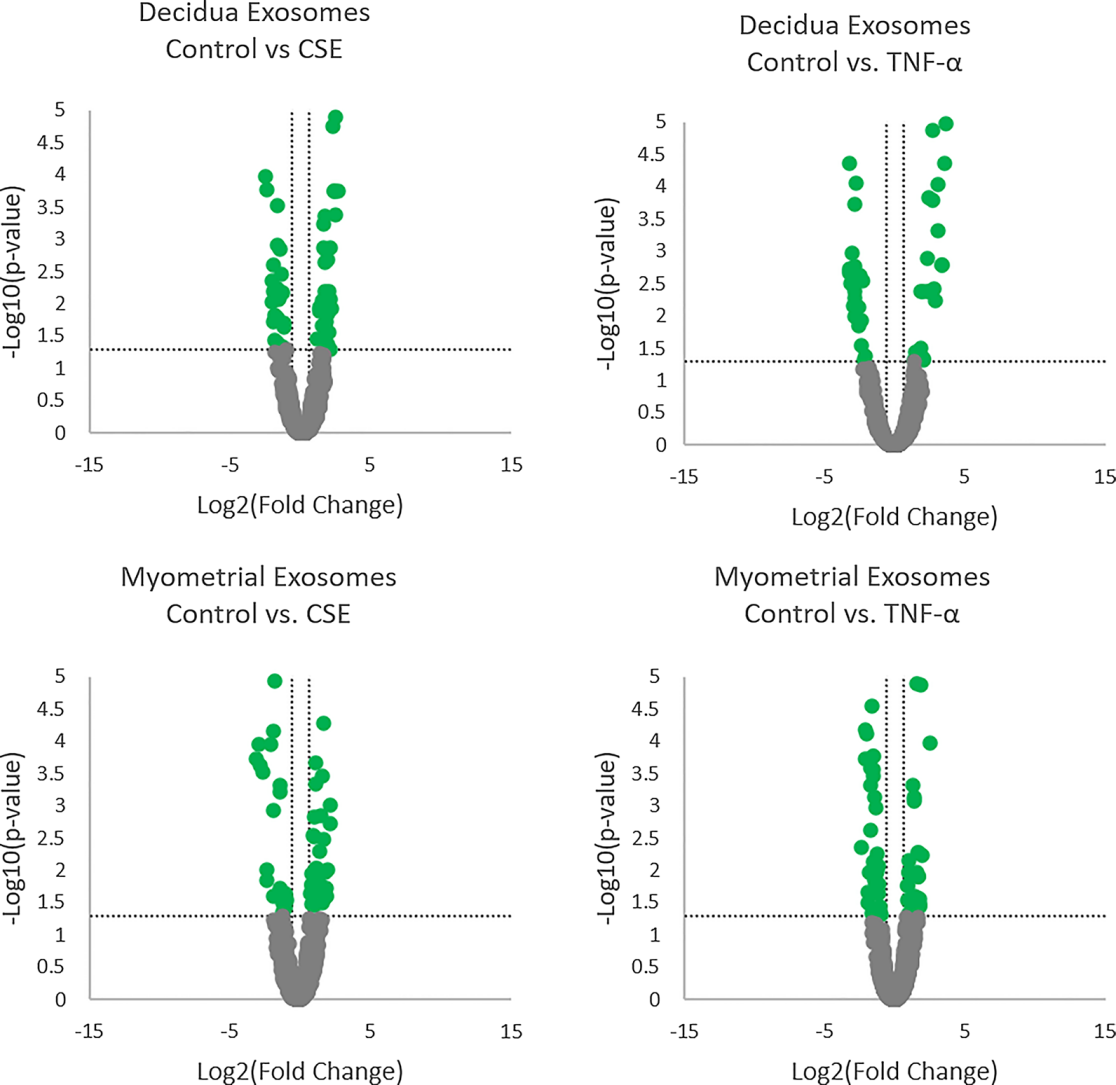
Volcano plots of differentially expressed exosomal protein cargos showing the comparison between control and CSE treatment, or control and TNF-α treatment, in decidual and myometrial cell-derived exosomes

The variation in the relative abundance of exosomal proteins between CSE and TNF-α groups, when compared to the control, for both decidual and myometrial exosomes is presented as a volcano plot (Fig. 4). The horizontal axis represents the log^2^ of the fold change and the vertical axis represents the *P* value. The horizontal dotted line shows *P* = 0.05. Each dot represents an identified protein, with the dots on the right being upregulated and the dots on the left being downregulated.

### Maternal exosomes display distinct pathways in response to different stimuli

**Fig. 5:**
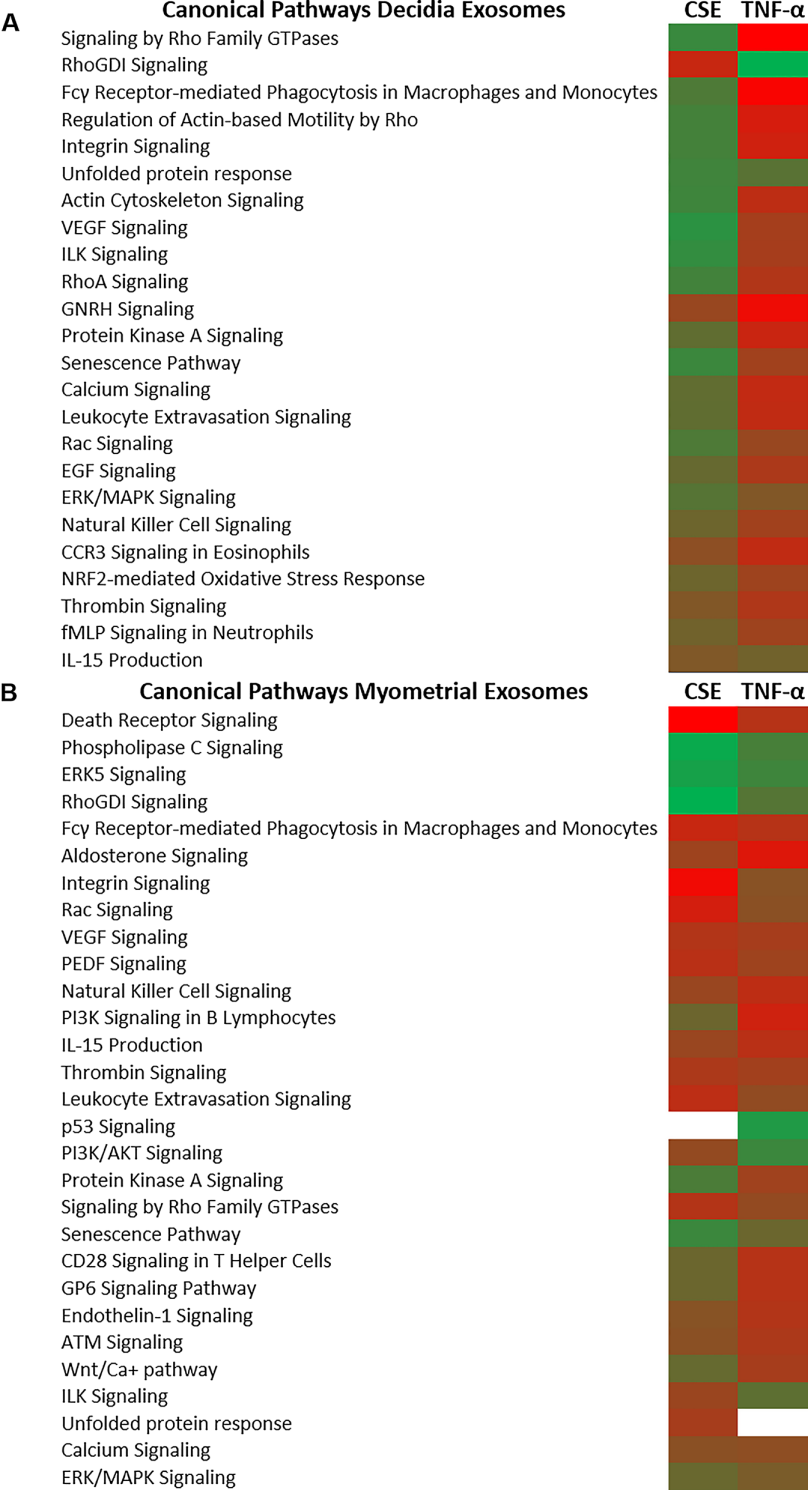
Ingenuity pathway analysis (IPA) of differentially regulated proteins depicts canonical pathways associated with each treatment. Canonical pathways are associated with decidual exosomes (**A**) or myometrial exosomes (**B**). Green represents upregulation and red represents downregulation compared to untreated controls

Differentially expressed exosomal proteins from each cell type under CSE and TNF-α conditions were further clarified for functional specificity. As shown in the heat maps, comparisons were made at multiple levels and pathways represented by proteomic cargo in exosomes from control, CSE or TNF-α treatment groups from both decidual and myometrial tissues were compared. Interestingly, very few pathways were commonly represented after CSE and TNF-α treatments in decidual cells. Upregulation of the unfolded protein response and downregulation of gonadotropin-release hormone (GNRH) and thrombin signaling was common after both treatments. However, multiple pathways were differentially represented in decidual exosomes, notably, senescence was upregulated by CSE but downregulated by TNF-α. Authenticity to differential expressed decidual exosome proteins and their pathway representation was seen with signaling by Rho Family GTPases that was upregulated by CSE and downregulated by TNF-α in decidual exosomes. Conversely, signaling by RhoGDI, a down-regulator of Rho family GTPases, was downregulated by CSE and upregulated by TNF-α. Multiple other pathways that represent a state of sterile inflammation were predominant in decidual exosomes following CSE treatment compared to TNF-α treatment (Fig. 5A).

Regardless of the direction of regulation, a number of common pathways were increased in myometrial exosomes after CSE and TNF-α treatments compared to decidual exosomes under the same conditions. Notable differences were seen in p53 and PI3/AKT signaling induction by TNF-α, and in Protein kinase A and senescence pathway induction by CSE (Fig. 5B). In summary, proteomics analysis suggests that exosomes reflect very distinct responses by decidual and myometrial cells to CSE and TNF-α treatment, and recipients of these exosomes may respond with specific functional effects.

After characterization of exosomes and confirmation of a differential presence of cargo protein, we tested the effects of maternal uterine cell exosomes on fetal membrane cells.

### Localization of maternal exosomes by fetal cells

**Fig. 6:**
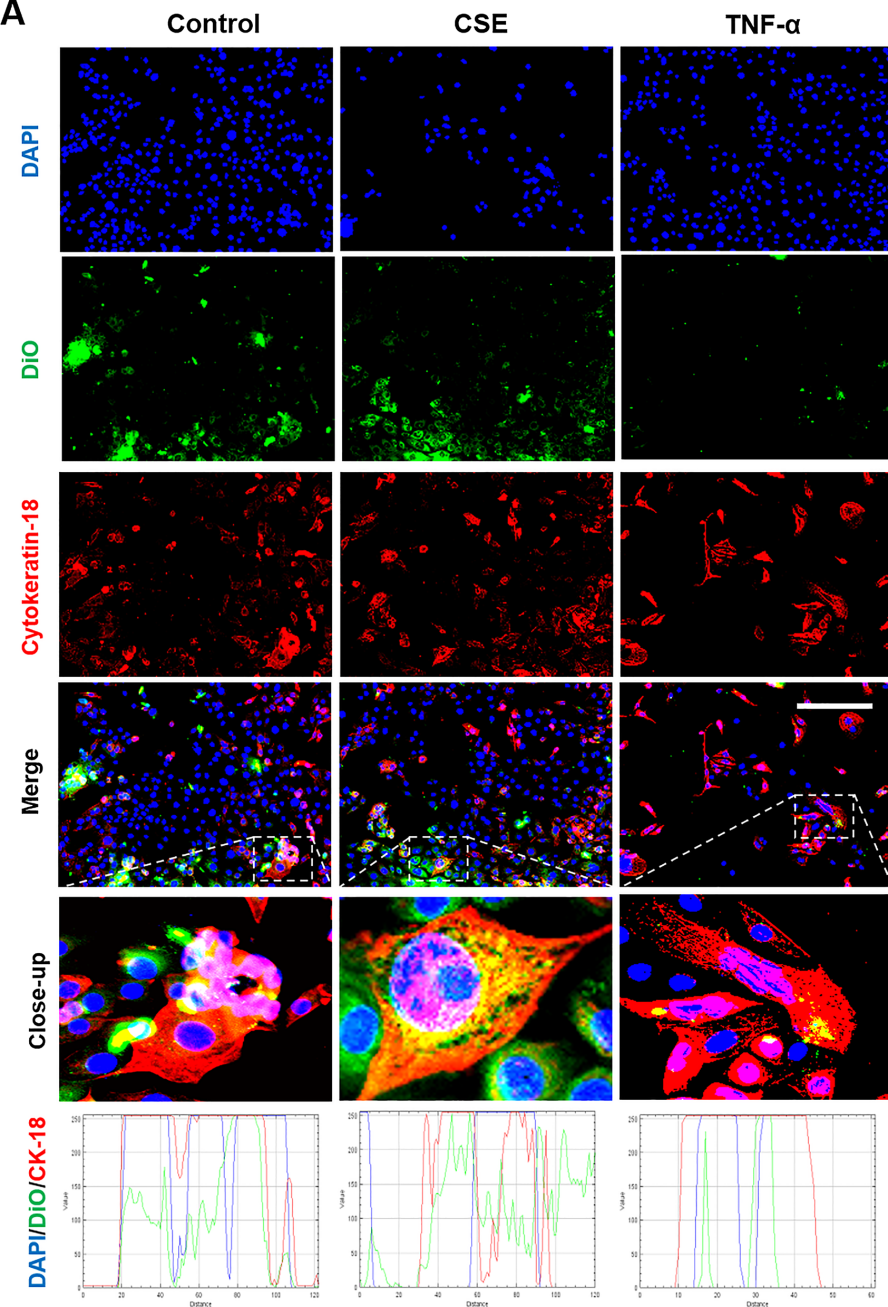

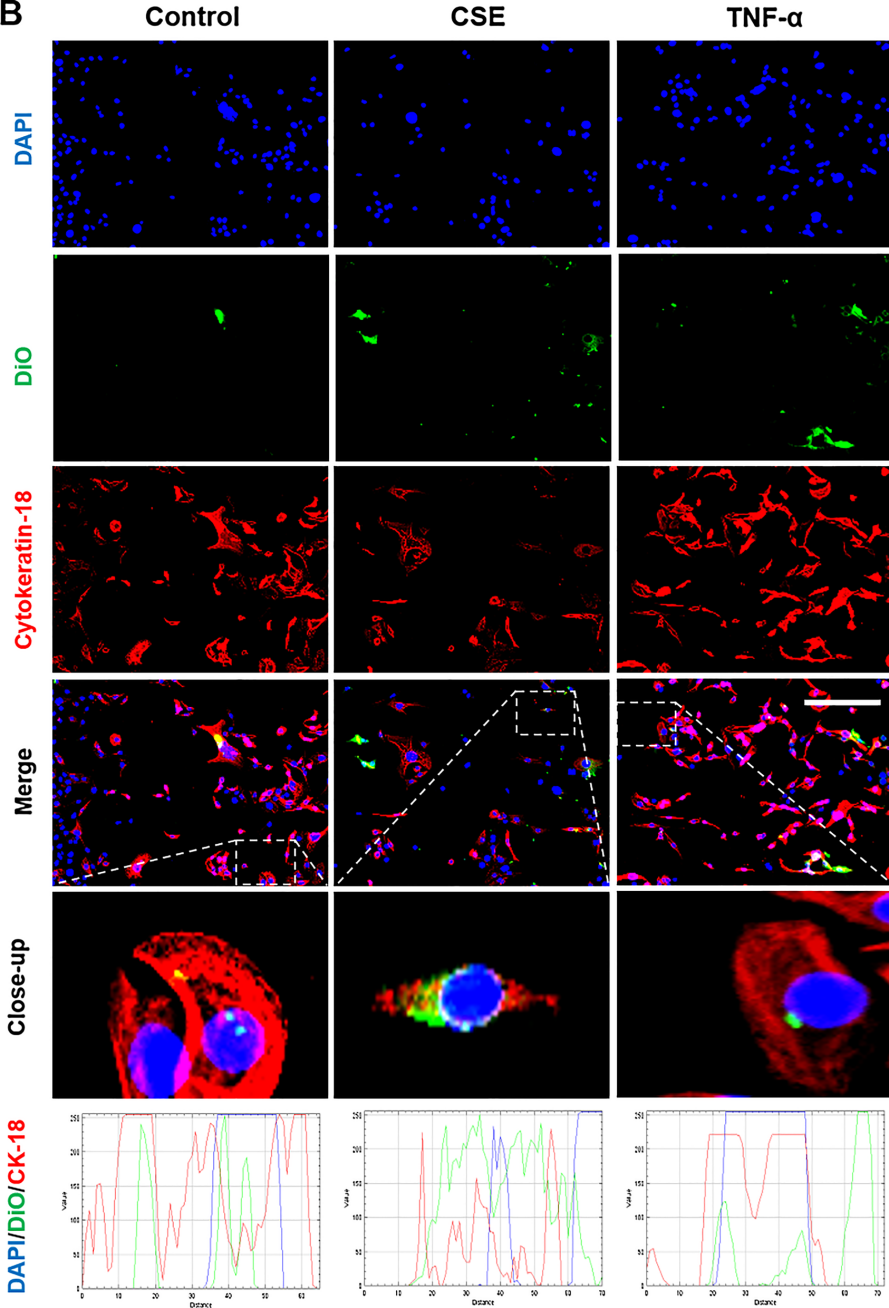
Immunocytochemical staining for exosome uptake by AECs. Representative images showing the AECs taking up DiO-labeled (green) decidual (**A**) and myometrial (**B**) cell-derived exosomes (106 exosomes per 50,000 cells) after each treatment, and the co-localization of DiO with cytoplasmic marker protein cytokeratin-18 (red) 4 h after each treatment. DAPI (blue) detects cell nuclei. The line graphs show overlap between DiO, CK-18 and DAPI in the cells. Close-up images are shown in the insets. Scale bar = 100 µm

**Fig. 7:**
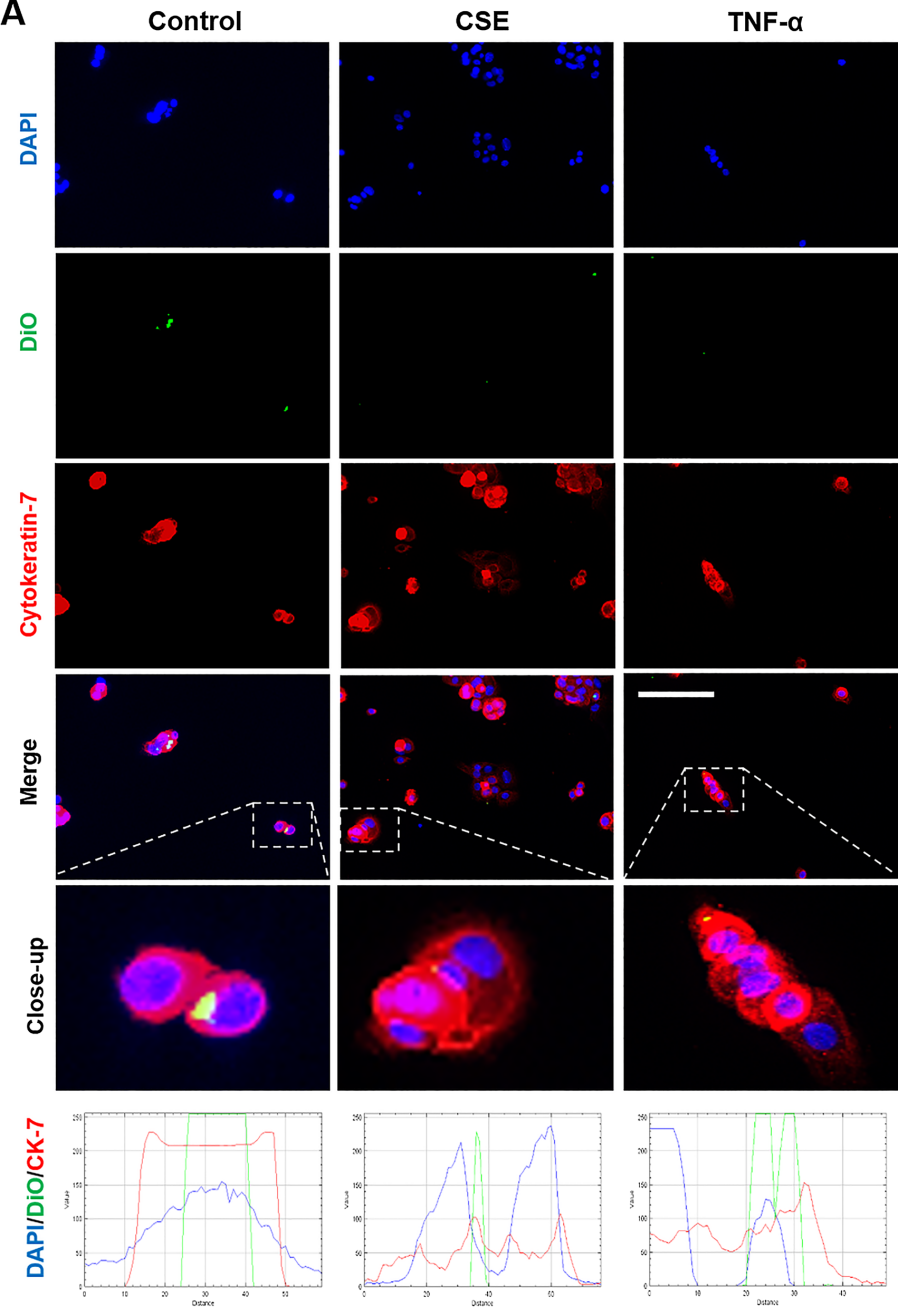

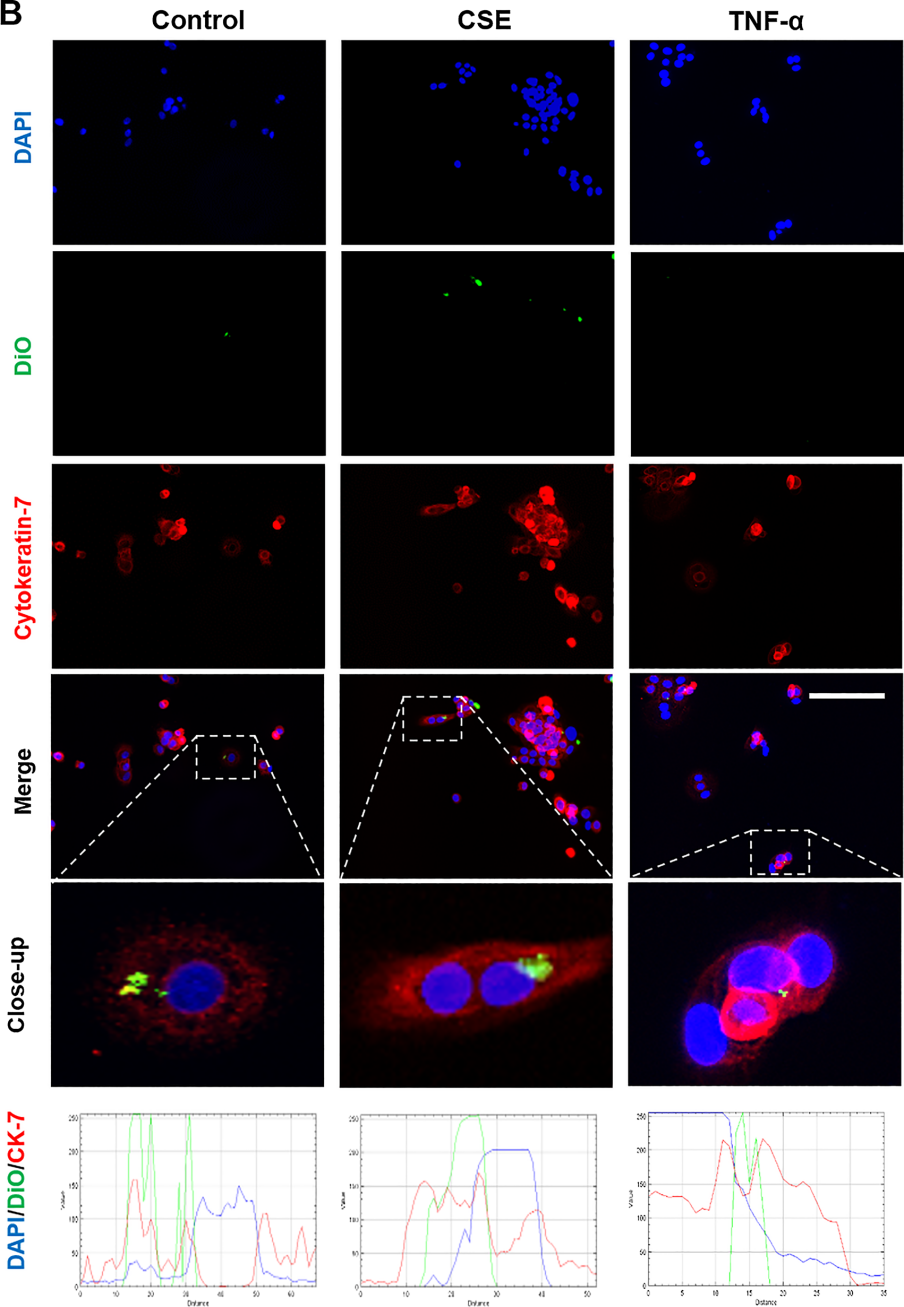
Immunocytochemical staining for exosome uptake by CTCs. Representative images showing the CTCs taking up DiO-labeled (green) decidual (**A**) and myometrial (**B**) cell-derived exosomes (106 exosomes per 50,000 cells) after each treatment, and the co-localization of DiO with cytoplasmic marker protein cytokeratin-7 (red) 4 h after each treatment. DAPI (blue) detects cell nuclei. The line graphs show overlap between DiO, CK-7 and DAPI in the cells. Close-up images are shown in the insets. Scale bar = 100 µm

Fluorescence microscopy and z-stack analysis was used to localize exosomes in recipient AECs and CTCs. As shown in Figs. 6 and 7, DiO labeled control, CSE and TNF-α group exosomes were detected within recipient cells. The location of the exosomes within cells, as opposed to adjacent to the cells, was confirmed using z-stack analysis and 3D reconstructions (Additional file 1: Figure S1).

### Maternal exosomes induce pro-inflammatory responses in AECs and CTCs

To confirm exosome-specific stimulation, ELISA was performed on media samples from the exosome blocking experiments. Incubation of cells in the cold led to a decrease in production of all cytokines by both AECs and CTCs. This suggests that exosome entry into these cells was blocked due to reduced endocytosis at 4 °C and supports the presence of an exosome-mediated cytokine response. Three cytokines, IL-6, TNF-α (both pro-inflammatory) and IL-10 (anti-inflammatory), were measured in media samples from AECs and CTCs. All concentrations are in pg/ml and significant *P* values are < 0.05. IL-10 was not measurable in AECs and is, therefore, not shown here.

**Fig. 8:**
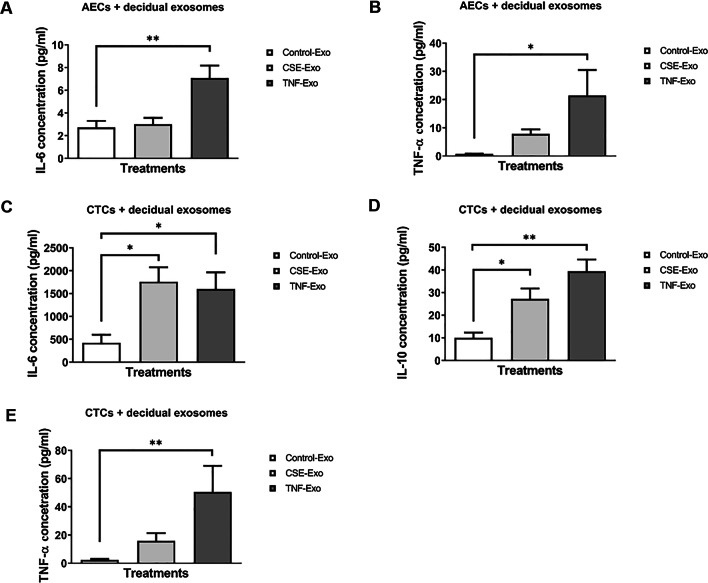
Cytokine analysis of the cell culture media by ELISA after incubation with control, CSE- and TNF-α-treated decidual cell-derived exosomes. **A**, **B** Detection of AEC-produced IL-6 and TNF-α in the media, 24 h after each treatment (N = 7). **C**–**E** Detection of CTC-produced IL-6, IL-10 and TNF-α in the media, 24 h after each treatment (N = 7). Data represented as mean ± SEM. *P < 0.05, **P < 0.01, one-way analysis of variance (ANOVA)

As shown in Fig. 8A and B (Additional file 2: Table S1), CSE- and TNF-α-treated decidual exosomes produced distinct responses in AECs. Both IL-6 and TNF-α release by AEC cells was increased when exposed to TNF-α-treated decidual exosomes, compared to when they were exposed to exosomes from control (untreated) decidual cells. In CTCs, both CSE- and TNF-α-treated decidual exosomes increased IL-6 production (Fig. 8C). CSE exosomes induced a fivefold increase in TNF-α release compared to control cell exosomes, but the data was not statistically significant. However, inflammatory cytokine-primed decidual exosomes significantly increased TNF-α output from CTCs (Fig. 8E). The increase in IL-10 was significantly greater in CTCs when exposed to either CSE or TNF-α exosomes, compared to control exosomes (Fig. 8D).

**Fig. 9:**
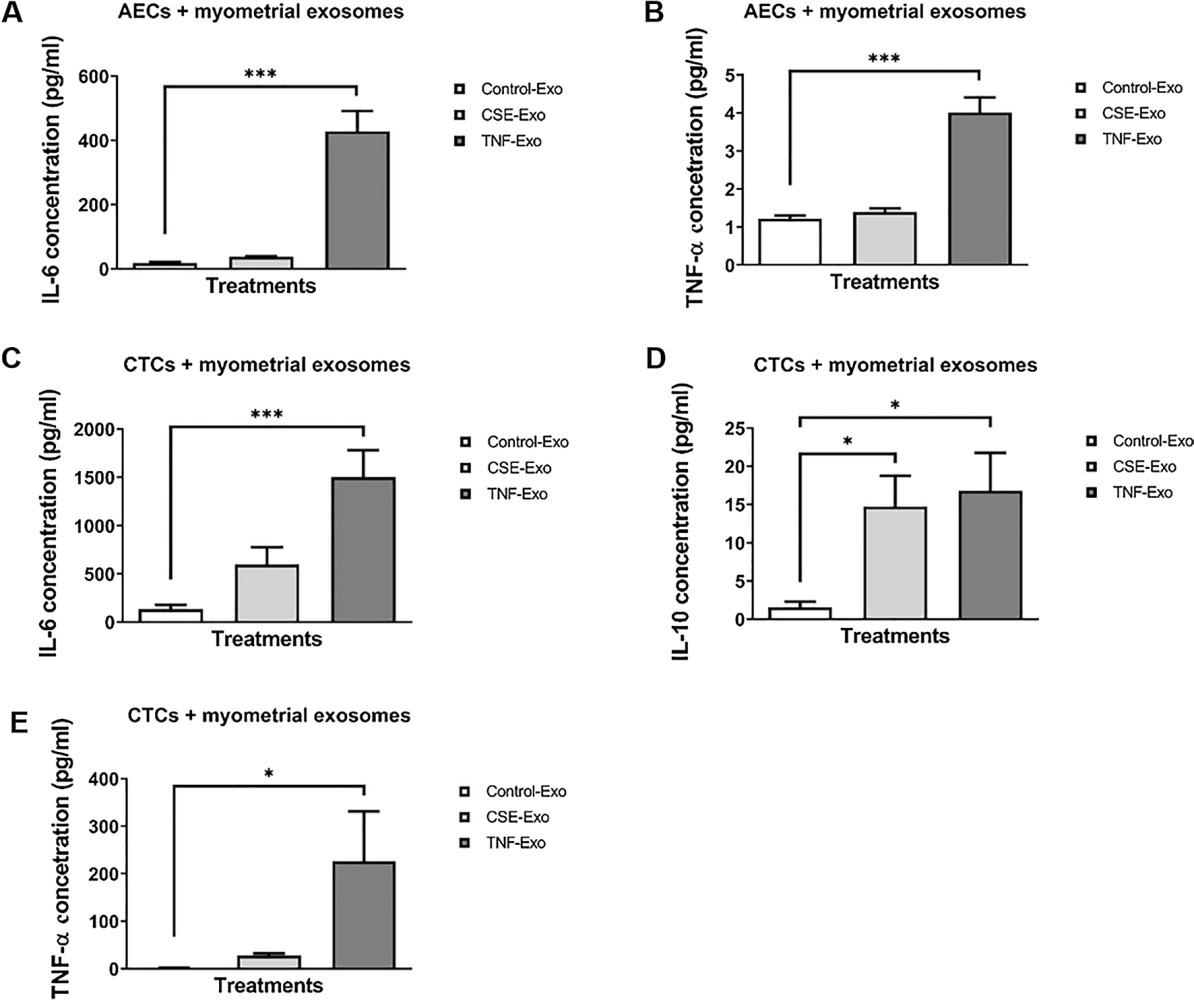
Cytokine analysis in the cell culture media by ELISA after incubation with control, CSE- and TNF-α-treated myometrial cell-derived exosomes. **A**, **B** Detection of AEC-produced IL-6 and TNF-α in the media, 24 h after each treatment (N = 7). **C**–**E** Detection of CTC-produced IL-6, IL-10 and TNF-α in the media, 24 h after each treatment (N = 7). Data represented as mean ± SEM. *P < 0.05, ***P < 0.001, one-way analysis of variance (ANOVA)

Myometrial cell-derived exosomes produced similar responses from AECs and CTCs (Fig. 9A–E, Additional file 2: Table S1), except that IL-6 production was significantly higher in CTCs when treated with exosomes from either CSE and TNF-α groups, compared to the control group (Fig. 9C). In summary, maternal uterine cells exposed to OS and inflammation produce exosomes with distinct cargo that can induce an inflammatory response in fetal cells.

## Discussion

Maternal–fetal communication plays an integral role in pregnancy maintenance and in determining timing of parturition. Prior reports have shown that different conditions experienced by the fetus result in fetal exosomes that can have a functional impact on the maternal side [24]. These studies have shown that inflammatory amplification in decidua and myometrium [11] and injection of these exosomes caused preterm birth by invoking NF-kB activation in the decidua, myometrium and cervix [24]. Although maternal exosomes can reach the fetal side, their role on the fetal side with respect to pregnancy or parturition is unclear [26]. Therefore, this study determined the functional role of maternal uterine cell-derived exosomes on the fetal side. Our principle findings are as follows: (1) the quantity and characteristics (size, shape, and markers) of CSE- and TNF-α-treated decidual and myometrial cell-derived exosomes were not different to control cells; (2) proteomic cargo contents of exosomes from treated and untreated cells had several similarities, however, certain unique proteins in these exosomes represented stimulus-specific responses and pathways; (3) maternal uterine cell exosomes are taken up by fetal membrane cells; (4) compared to exosomes from control decidual and myometrial cells, stimulated cell-derived exosomes induced pro-inflammatory responses in AECs and CTCs; and (5) increased IL-10 release by CTCs suggest a differential immune response by various cell types in the fetal membranes. In summary, various pathophysiological conditions cause maternal exosomes to carry inflammatory mediators that can result in cell type dependent fetal inflammatory response.

Inflammatory responses by stimulated decidual and myometrial exosomes on AECs and CTCs showed a differential effect when we report the stimulus (exposure) and the recipient cell dependent cytokine response. AECs did not produce detectable levels of IL-10, as AECs are a poor source of IL-10 in the membranes, whereas CTCs are major producers of IL-10 [41]. This is partly because CTCs are a major barrier between membranes and the decidua, and partly because they produce high levels of progesterone and IL-10 to reduce inflammatory responses or immune cell infiltration from the maternal side to protect the membranes [42]. Even though AECs and CTCs respond with an increase in pro-inflammatory cytokines, CTCs tend to balance a pro-inflammatory response with an increased production of IL-10. Although an increase in IL-10 alone is not an indicator of balanced inflammatory response, it demonstrates tendencies of different fetal membrane cell compartments to regulate inflammation in response to maternal exosomes carrying various stimuli.

Both physiological and pathological communications can be mediated between the mother and the fetus via exosomes. Since all uterine cells constantly secrete exosomes, their cargo is likely involved in maintaining balanced tissue functions [43, 44]. Pathological changes to cells, in response to endogenous or exogenous factors during pregnancy, produce exosomes that are capable of causing a parturition-associated fetal inflammatory state through delivery of pro-inflammatory mediators [24]. OS-inducing factors are often encountered by the mother during pregnancy, contributing to adverse pregnancy outcomes [43, 44]. Behavioral risk factors (drug or alcohol abuse and cigarette smoking), obesity, malnutrition, environmental pollutants, and infections can generate OS responses in uterine cells. Inflammation of the uterine cavity can arise from infectious and noninfectious conditions [45–48]. OS-induced tissue damage is one of the major sources of noninfectious inflammation in the feto-maternal tissues [36, 49, 50]. By incorporating two stimuli that represent OS and inflammation in this study, we determined that changes in maternal tissue (patho)physiology can impact fetal well-being in utero, with an increase in inflammation caused by exosome-mediated paracrine signaling. The clinical relevance of this work is important to understand feto-maternal communication involved in both term and preterm parturition. In response to maternal exosomal messaging systems, the fetus can respond by generating various biochemical signals that may either help to maintain pregnancy by balancing inflammation, or by increasing inflammatory mediators that can signal parturition-associated changes. As shown previously, a fetal inflammatory response can be conveyed to the mother through pro-inflammatory cargo-enriched exosomes that can trigger preterm birth [24]. Based on these reports, we postulate that maternal risk factors, although clinically asymptomatic on the maternal side, can trigger potential labor-inducing changes on the fetal side.

Exosomes are beneficial in perinatal medicine in multiple ways: (1) exosome trafficking, and changes induced at specific sites, help to understand their functional role under certain conditions [22]; (2) as exosomes are inert, non-immunogenic and can cross feto-maternal barriers, they can be engineered to contain specific cargo molecules, such as drugs to address various pregnancy pathologies [51]; and (3) exosomes are potential biomarkers as they carry snapshots of the physiological state of the cell they originated from [8, 52, 53]. As reported here, the functional contribution of exosomes derived from maternal uterine cells exposed to specific conditions has now been established. This study has shown that paracrine signaling by the mother can generate a fetal inflammatory response and, therefore, can contribute to the design of strategies to reduce the risk of adverse pregnancy outcomes. We have recently reported that feto-maternal inflammation, primarily mediated by fetal inflammatory responses to infection, is a key trigger mechanism for preterm birth in animal models. Injection of engineered exosomes that contained pro-transcription factor NF-kB inhibitor, delayed infection-induced preterm birth. This delay was associated with a reduction in fetal inflammatory responses, indicated by a reduction in NF-kB activation, histologic chorioamnionitis, and inflammatory cytokines in various fetal biologic compartments. Understanding the feto-maternal initiators and effectors of preterm birth in response to various risk factors is critical in providing tailored interventions.

The use of exosomes as biomarkers is beneficial for indicating pregnancies that have a high risk status [8, 52, 53]. In pregnancy, the first report on exosomes as biomarkers was reported by McElrath’s group, where first-trimester maternal plasma microparticle proteomic cargo with an inflammatory signature was found to carry a high risk for preterm delivery < 34 weeks [54]. Recent work by the same group has reported that circulating microparticle cargo proteins during the first trimester may indicate a risk of developing preeclampsia [55]. In addition, various reports have shown that placental exosomes (fetal) that are positive for placental alkaline phosphatase (PLAP) isolated from maternal plasma are a useful biomarker for the development of preeclampsia [54–57]. The presence of PLAP-positive exosome cargo has also been reported in spontaneous preterm birth [17, 25]. Understanding the mechanisms that generate specific cargo-enriched exosomes by specific cells in response to various tissue environments, and their functional roles, can further highlight the potential of these exosomes as biomarkers for predicting risk.

As mentioned in the introduction, the class of microparticles includes exosomes, but also includes larger vesicles (microvesicles that may be different to exosomes) that can carry biochemicals [8, 22]. This disparity is due to differences in biogenesis mechanisms and facilitators of cargo packaging. Although overlap exists between sizes of microvesicles and exosomes, the predominant class of vesicles in a preparation depends on the protocol used for isolation [58]. We have not yet examined the role of decidual, and myometrial cell-derived microvesicles. It is likely that maternal exosomes are not alone in producing a specific response on the fetal side. Microvesicle cargo may either provide additive effects or may try to balance the impact of exosomes. These are topics for future studies and hence, a limitation of this report. We have also not examined the effect of maternal exosomes on the placental side, or the effect of other maternal tissues (e.g. cervical) on the fetal side. Functional validation using animal models is also necessary prior to establishing a precise role for maternal exosomes on fetal tissues. Regardless of these limitations, this study provides a platform for similar future studies.

## Conclusion

This is the first study to isolate and characterize maternal decidual and myometrial cell-derived exosomes. Maternal exposure to various risk factors during pregnancy, specifically those causing oxidative stress and inflammation, can generate exosomes with distinct cargo proteins that can cause a fetal inflammatory response. Chorion trophoblast cells at the maternal–fetal interface may function as a barrier for maternal exosomal function on the fetal side.

## Supplementary Information

**Additional file 1. Figure S1**. 3D reconstructions of exosome internalization using ImarisViewer on z-stack images taken by Keyence microscope. Uptake of control, CSE- and TNF-α-treated decidual (**A**) and myometrial (**B**) cell-derived exosomes by AECs. Uptake of control, CSE- and TNF-α-treated myometrial (**C**) cell-derived exosomes by CTCs.

**Additional file 2. Table S1**. Cytokine concentrations in AEC and CTC cells treated with control, CSE- and TNF-α-treated exosomes from both decidual and myometrial cells (N=7).

## Data Availability

Information related to data availability included in the methods section.

## Abbreviations

AEC: Amnion epithelial cells
AGC: Automatic gain control
BPE: Bovine pituitary extract
BSA: Bovine serum albumin
CAN: Acetonitrile
CSE: Cigarette smoke extract
CTC: Chorion trophoblast cells
DAPI: 4′,6-Diamidino-2-phenylindole
DDA: Data-dependent acquisition
DMEM: Dulbecco’s Modified Eagle Medium
ELISA: Enzyme-linked immunosorbent assay
EV: Extracellular vesicles
FBS: Fetal bovine serum
IL: Interleukin
IPA: Ingenuity pathway analysis
LC–MS/MS: Liquid chromatography-tandem mass spectrometry
MIPS: Monoisotopic precursor selection
MS: Mass spectrometry
OS: Oxidative stress
PLAP: Placental alkaline phosphatase
TCEP: Tris(2-carboxyethyl)phosphine
TEAB: Triethylammonium bicarbonate
TNF: Tumor necrosis factor
