# Supplementary figures and images for: Extracellular vesicles from maternal uterine cells exposed to risk factors cause fetal inflammatory response
Source: Cell Commun Signal. 2021 Oct 7;19:100. doi: 10.1186/s12964-021-00782-3 (PMC8499538; doi:10.1186/s12964-021-00782-3)

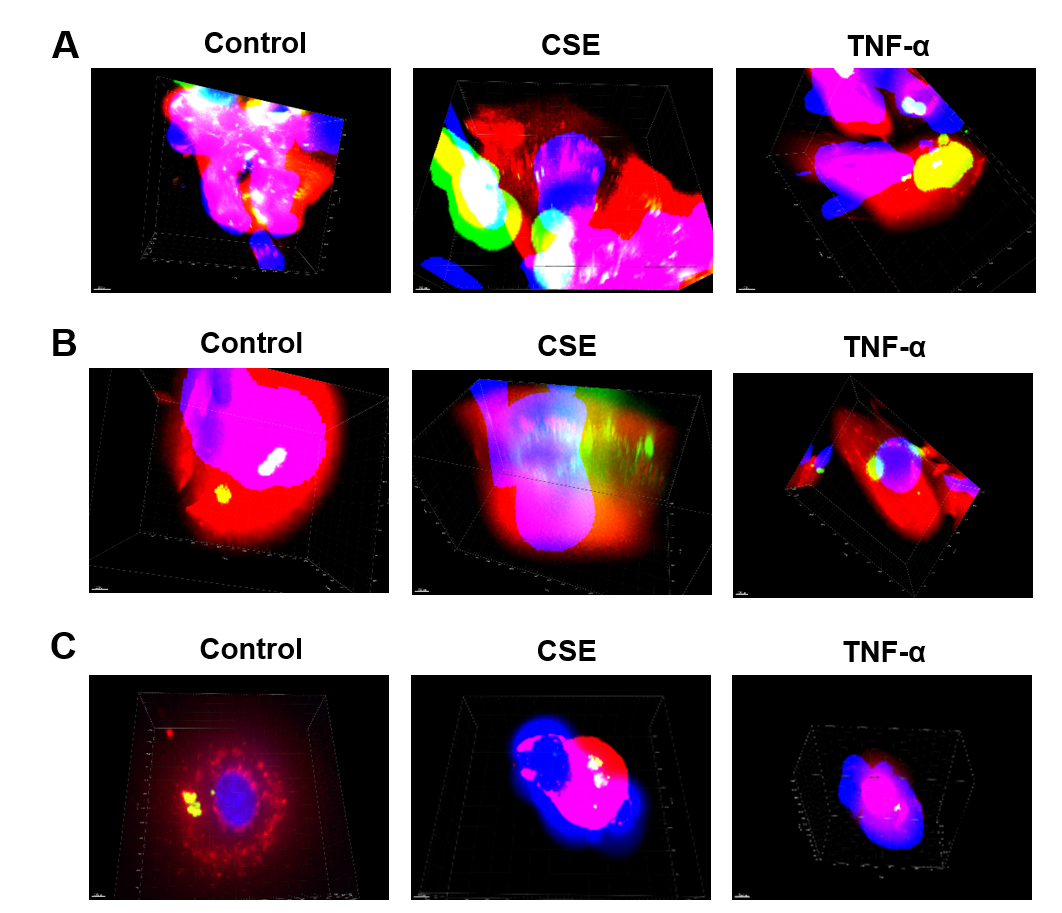

Supplement: Supplementary file 2 — Additional file 1. Figure S1. 3D reconstructions of exosome internalization using ImarisViewer on z-stack images taken by Keyence microscope. Uptake of control, CSE- and TNF-α-treated decidual (A) and myometrial (B) cell-derived exosomes by AECs. Uptake of control, CSE- and TNF-α-treated myometrial (C) cell-derived exosomes by CTCs. [file 12964_2021_782_MOESM2_ESM.tif]

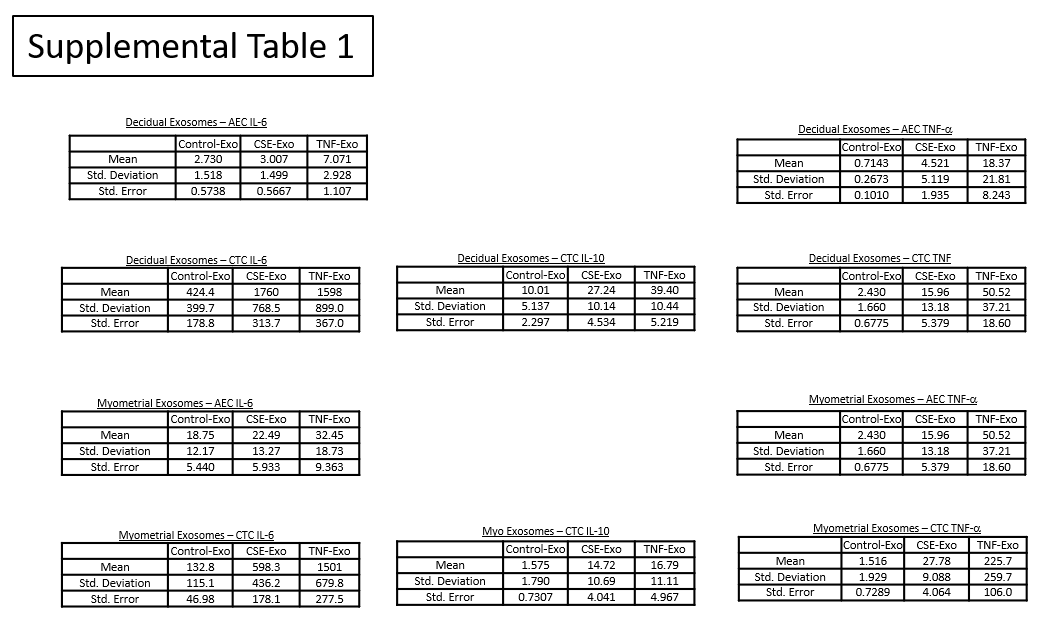

Supplement: Supplementary file 3 — Additional file 2. Table S1. Cytokine concentrations in AEC and CTC cells treated with control, CSE- and TNF-α-treated exosomes from both decidual and myometrial cells (N=7). [file 12964_2021_782_MOESM3_ESM.tif]
